# Supplementary material for: Health Education about Lifestyle-Related Risk Factors in Gynecological and Obstetric Care: A Qualitative Study of Healthcare Providers’ Views in Germany
Source: Int J Environ Res Public Health. 2022 Sep 16;19(18):11674. doi: 10.3390/ijerph191811674 (PMC9517227; doi:10.3390/ijerph191811674)
Supplement: Supplementary file 1 [file ijerph-19-11674-s001.zip › ijerph-1904715-supplementary.pdf]

Causal Conditions (conditions that lead to the development of a phenomenon)	Health education about LRRFs depends on a woman's medical history and specific needs, her SES and whether she has a migration background.
Context (the set of characteristics in which a phenomenon is embedded)	Health education about LRRFs is context dependent. In inpatient healthcare settings, education about LRRFs is of lower priority as compared to outpatient healthcare settings. Health education about LRRFs takes place at the initial consultation, often before the 10th week of pregnancy. The time taken for health education about LRRFs varied between 5 minutes and 1 hour.
Intervening Conditions (general conditions that influence action strategies)	Central barrier to educate women about LRRFs is the lack of standardized guidelines, and there are no standardized procedures and documents applied. Available time to educate women about LRRFs during pregnancy and lactation is limited due to the billing options of the health insurance companies. There is an unclear assignment of responsibilities regarding education about LRRFs during pregnancy and lactation.
Actions Strategies (an action that is performed or not to overcome a phenomenon)	HCPs applied a demand-driven healthcare approach regarding education about LRRFs, e.g. specific information in case of special diet. HCPs opinion on whether there are differences between health education during pregnancy and lactation are inconsistent.
Consequences of Current Practice (real or hypothetical consequences of a phenomenon)	Education of women on LRRFs during pregnancy and lactation should take place prior to conception, across diverse settings. Support should be provided by other health organizations, concerning billing possibilities and education about LRRFs during pregnancy.

Supplement Table S1: Summary of Results.
